# Association of Catechol-*O*-methyltransferase single nucleotide polymorphisms, ethnicity, and sex in a large cohort of fibromyalgia patients

**DOI:** 10.1186/s41927-018-0045-4

**Published:** 2018-12-12

**Authors:** Chee Lee, Ginevra Liptan, Svetlana Kantorovich, Maneesh Sharma, Ashley Brenton

**Affiliations:** 1 Proove Biosciences, Inc., Irvine, CA USA; 2 The Frida Center for Fibromyalgia, Portland, OR USA; 3 Interventional Pain Institute, Baltimore, MD USA; 4 Mycroft Bioanalytics, Inc., 299 South Main Street, Suite 2300, Salt Lake City, UT 84111-2278 USA

**Keywords:** Fibromyalgia, Chronic pain, Precision medicine, Genomics, Catechol-O-methyltransferase

## Abstract

**Background:**

Fibromyalgia (FM) is a complex, centralized pain condition that is often difficult to diagnose and treat. FM is considered to have a genetic background due to its familial aggregation and due to findings from multiple candidate-gene studies implicating catecholaminergic and serotonergic neurotransmitter systems in chronic pain. However, a multi-factorial analysis of both genetic and environmental risk factors is lacking. A better characterization of the interplay of risk factors may assist in understanding the pathophysiology of FM, its clinical course, and assist in early diagnosis and treatment of the disorder.

**Methods:**

This retrospective study included 60,367 total participants from 237 clinics across the USA. Of those, 2713 had been diagnosed with fibromyalgia, as indicated by ICD code. Logistic regression was used to test for associations of diagnosed FM in study subjects with *COMT* SNPs and *COMT* haplotypes, which were previously found to be linked with pain sensitivity, as well as demographics such as age, sex, and ethnicity. The minor allele frequencies of *COMT* SNPs in the FM population were compared with 1000 Genomes data using a χ2 test to determine significant deviations from the estimated population allelic frequencies.

**Results:**

FM diagnosis was strongly associated with sex, age, and ethnicity. Females, those between 49 and 63 years, and non-Caucasians were at higher risk of FM. Females had 1.72 increased odds of FM (*p* = 1.17 × 10^− 30^). African-Americans were 1.52 times more likely to have a diagnosis of FM compared to Caucasians (*p* = 3.11 × 10^− 12^). Hispanics were less likely to have a diagnosis of FM compared to Caucasians (*p* = 3.95 × 10^− 7^). After adjusting for sex and ethnicity, those in the low age group and mid age group had 1.29 *(p* = 1.02 × 10^− 5^) and 1.60 (*p* = 1.93 × 10^− 18^) increased odds of FM, respectively, compared to the high age group, where age was categorized by tertile (low (< 49), mid (49–63), and high (> 63)). The *COMT* haplotypes associated with pain sensitivity were not associated with FM, but African-Americans were 11.3 times more likely to have a high pain sensitivity *COMT* diplotype, regardless of FM diagnosis. However, the minor alleles of *COMT* SNPs *rs4680*, *rs4818*, *rs4633* and *rs6269* were overrepresented in the FM population overall, and varied when compared with ethnically-similar populations from 1000 Genomes.

**Conclusions:**

This is the largest study, to date, that examines demographic and genetic associations of FM in a diverse population. While pain sensitivity-associated *COMT* haplotypes were not found to be directly associated with FM diagnosis, the minor alleles that make up the *COMT* haplotypes were overrepresented in the FM population, suggesting a role of *COMT* in FM. Future studies are needed to elucidate the exact role of *COMT* variation in widespread pain conditions, such as FM. Clinically, this information can be used to provide insight into the pathways underlying FM and to identify those at greater risk of developing FM.

**Electronic supplementary material:**

The online version of this article (10.1186/s41927-018-0045-4) contains supplementary material, which is available to authorized users.

## Background

Fibromyalgia (FM) is a chronic illness that affects an estimated 5.4% of the US population, and is 2.3 times more prevalent in women than men [1]. FM is characterized by widespread pain, tenderness and fatigue, and is notoriously challenging to diagnose and treat. Patients suffering from FM report a low quality of life [2], and are 3.4 times more likely to suffer from major depressive disorder than those without FM [3].

FM is difficult to diagnose and treat because it manifests a diverse range of symptoms that affect multiple body systems, and it is frequently accompanied by comorbid disease. FM is often a diagnosis of exclusion after other possible conditions have been treated without success and ruled out. Common FM symptoms include pain sensitivity, chronic fatigue, muscle tenderness, mental fog, sleep disturbances, and digestive issues. These same symptoms are also reported in other illnesses, such as temporomandibular disorder, irritable bowel syndrome, and mental disorders [4, 5]. In fact, FM co-occurs with these conditions frequently [6]. FM is also often associated with other diseases like hypothyroidism [7], rheumatoid arthritis, and systemic lupus erythematosus [8], as well as psychiatric diseases, such as major depressive disorder, and mood and anxiety disorders [4]. Current diagnostic criteria for FM rely solely on subjective measures, as there is currently no accepted objective testing. The 1990 ACR diagnostic criteria require presence of pain upon pressure applied to at least 11 of 18 specific tender points, and the updated 2010 criteria use a symptom survey as a diagnostic tool. The development of an objective genetic biomarker would significantly improve our ability to diagnose fibromyalgia.

The multi-system pathology of FM stems in part from autonomic nervous system dysfunction, in particular excess sympathetic activity which has been well demonstrated in heart-rate variability studies [9, 10]. Although incompletely understood, abnormal catecholaminergic signaling has been identified in the pathogenesis of FM and central sensitization [11]. COMT genetic variations that result in excess levels of catecholamines may promote development of chronic pain via stimulation of adrenergic receptors in the peripheral and central nervous systems [12, 13]. The autonomic nervous system likely plays a role in regulation of fibromyalgia pain, as functional MRIs in FM subjects with higher levels of sympathetic nervous system activity demonstrated more temporal summation of pain [14]. Variations in COMT activity are one factor that may contribute to the complex interaction of autonomic nervous system and pain regulation.

Catecholamines (i.e. dopamine, epinephrine, norepinephrine) are key neurotransmitters in both pain-inhibiting and pain-initiating pathways, depending on gene expression factors, and cell type [15, 16]. Several gene-candidate studies have examined serotonergic, dopaminergic, and catecholaminergic markers as major players in central sensitization and FM pain [17–29] . In particular, the missense variant *val*^*158*^*met* in catecholamine-O-methyltransferase gene (*COMT*) single nucleotide polymorphism (SNP), *rs4680*, results in significantly reduced catecholaminergic turnover, which has been shown to be associated with FM [22–29]. However, some studies in non-Caucasian populations have conflicting results [30, 31], showing only weak association of *rs4680* with FM, or stronger association with other *COMT* SNPs, suggesting the association of COMT SNPs may be ethnic group specific.

Haplotypes consisting of *COMT* SNP *rs4680*, *rs6269*, *rs4633*, and *rs4818*, have also been shown to be associated with pain sensitivity [32, 33], although it is unclear if those haplotypes have a role in chronic widespread pain, like FM. Nicholl et al. [34] did not find a link with *COMT* haplotypes and FM in two separate cohorts comparing pain-free individuals to those with FM; however, both cohorts consisted of Caucasians and one cohort exclusively of men. Conversely, Martinez-Jauand et al. [24], in an age-matched case-control study of patients with British ancestry, found FM patients to more likely have *COMT* haplotypes corresponding to higher pain sensitivity. Studies of *COMT* in FM thus far have been limited to small cohorts and specific populations. The focus of our study was to evaluate both *COMT* polymorphisms and phenotypic associations with FM in order to identify defining factors in diagnosis. This is the largest study thus far to examine associations of *COMT* SNPs and *COMT* haplotypes with FM.

## Methods

This multi-center, observational clinical study (study protocols 2015/11/1, HS-15-00191, 2016/07/26, 1JUL14-62CR, 1,309,306, 20,152,748, 1JAN15–26, 1JAN15-20CR, 1JAN15-14CR) was reviewed, approved, and overseen by Solutions IRB, an institutional review board licensed by the United States Department of Health and Human Services, Office for Human Research Protections. All participants signed informed consent forms prior to data collection.

### Study participants

Participants in the study were recruited from 237 clinics across the US, including orthopedic, spinal, pain management, and internal medicine clinics. Recruitment also included the criterion that the administrating medical professional deemed the genetic test was medically necessary to assess risk of FM. In total, the study comprised of 60,367 participants. Among the study participants, a group of 2713 individuals with FM (a 4.5% diagnosis rate) were identified using the International Statistical Classification of Diseases and Related Health Problems (ICD)-9 series code 729.1 for “myalgia and myositis” and ICD-10 series code M79.7 for “fibromyalgia.” All participants in the study who had complete genetic data for the *COMT* single nucleotide polymorphisms (SNPs), *rs6269*, *rs4633*, *rs4818*, and *rs4680*, were included in downstream analysis. A control group of participants, referred to as non-FM, were identified as those who had not been diagnosed with FM via ICD codes. Non-FM participants were also filtered by ICD codes to exclude conditions that were comorbid with FM (such as osteoarthritis and temporomandibular joint disorders), as well as chronic conditions and pain diagnoses, to reduce the rate of undiagnosed FM. The non-FM group included 32,141 participants in total.

### DNA analysis

#### DNA sample collection and genotyping

The methods were described previously [35].

#### COMT haplotyping

Haplotypes for COMT enzymatic activity were identified according to Diatchenko, et al. [18]. The three haplotypes for *COMT* SNPs *rs6269*, *rs4633*, *rs4818*, and *rs4680* are as follows: high pain sensitivity haplotype (HPS) as A_C_C_G, average pain sensitivity (APS) as A_T_C_A, and low pain sensitivity (LPS) as G_C_G_G. LPS corresponds to higher COMT enzymatic activity, APS to average activity, and HPS to lower activity. To verify haplotypes, the expectation maximization algorithm implemented by the *haplo.em* function in the R package *haplot.stats* [36] was used to empirically compute maximum likelihood estimates of haplotype probabilities. Only subjects with the major diplotypes (LPS/LPS, LPS/APS, APS/APS, LPS/HPS, APS/HPS, and HPS/HPS) were included in the association testing. Among the *COMT* diplotypes, high pain sensitivity corresponds to HPS/HPS or APS/HPS diplotypes, and low pain sensitivity corresponds to an LPS/LPS diplotype.

### Statistical analysis

#### Associations with demographics

Logistic regression was used to model the log odds of FM compared to the non-FM group in association with sex, ethnicity, and age. To increase power and provide clinically relevant interpretations, age was re-categorized by tertile: low (< 49), mid (49–63), and high (> 63). All statistical analyses were performed in R version 3.2.5.

#### Genetic associations with FM

To reduce bias due to patient recruitment and enrollment in the study, the FM group was compared to individuals in the 1000 Genomes Project [37] from US populations (*n* = 224) to assess genetic associations with the *COMT* SNPs. This allowed testing for significant deviations of allele frequencies in the FM group compared to the general population. A *χ*^2^ test of proportions was used to determine if the observed minor allele frequencies (MAFs) in the FM group significantly deviated from the 1000 Genomes MAFs of the respective population with similar demographics. For *rs4680*, the minor allele corresponds to the *val*^*158*^*met* variant (major>minor: G > A). Minor alleles of the other *COMT* SNPs are *rs6269* (A > G), rs4633 (C > T), and rs4818 (C > G).

## Results

### Demographics FM study participants

**Fig. 1:**
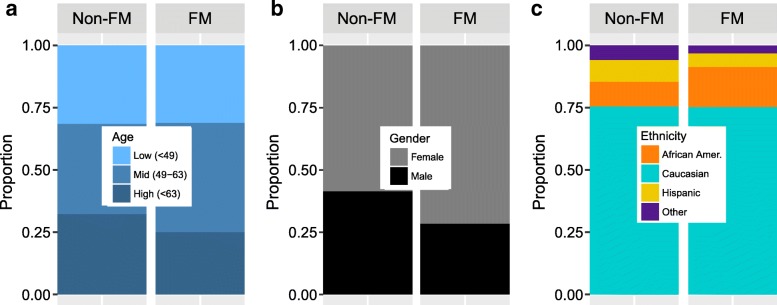
Distribution of FM diagnosis by (**a**) age, (**b**) sex, and (**c**) ethnicity. Those diagnosed with FM were more likely to be in the middle age tertile (49–63), female, and African-American compared to the non-FM group. Adjusted odds ratios associated with each specific demographic is shown in Table 1

FM was found to be significantly more common in certain demographics (Tables 1, 2, and Fig. 1). FM is well known to have a higher occurrence in women [38, 39], which was also observed in this study. The FM group (*n* = 2713) was comprised of 71.6% females compared with 58.5% females in the non-FM group (*n* = 32,141), which corresponded to a 1.72 increased odds of FM (*p* = 1.17 × 10^− 30^; Tables 1, 2 and Fig. 1). The overall average age of the study population was 55.6 years (standard deviation = 15.5). The average age in FM and non-FM groups were 55.7 and 54.9 years, respectively. Considering the wide distribution of ages in the study, age was re-categorized by tertile: low (< 49), mid (49–63), and high (> 63). On average, study patients in the low and mid age group had 1.29 *(p* = 1.02 × 10^− 5^) and 1.60 (*p* = 1.93 × 10^− 18^) increased odds of FM, respectively, compared to the high age group, after adjusting for gender and ethnicity (Table 2). Interestingly, the ethnicity associated with the highest diagnosis rate of FM was African-American. After adjusting for age group and sex, African-Americans were 1.52 times more likely to have a diagnosis of FM compared to Caucasians (*p* = 3.11 × 10^− 12^;Table 2 and Fig. 1). Hispanics were less likely to have a diagnosis of FM compared to Caucasians (*p* = 3.95 × 10^− 7^). There was no difference in the distribution of ethnicity or sex between the non-FM group and the group of study patients that were excluded from the non-FM group due to FM comorbidities and pain diagnoses.

**Table 1:** Demographics of study participants and US populations from the 1000 Genomes Project

Group	Mean Age	Ethnicity	*n* (% Total)	% Females
Fibromyalgia (FM)	54.9	All:	2713 (100%)	72%^a^
		African-American:	380 (14%)^b^	71%
		Caucasian:	1779 (66%)^b^	71%
		Hispanic:	130 (4.8%)	71%
		Other:	34 (1.3%)	65%
Non-Fibromyalgia (Non-FM)	55.7	All:	32,141 (100%)	59%
		African-American:	2863 (8.9%)^b^	65%
		Caucasian:	21,807 (68%)^b^	58%
		Hispanic:	2515 (7.8%)	57%
		Other:	2028 (6.3%)	60%
1000 Genomes (US populations)	*N/A*	All:	224 (100%)	52%
		African-American:	61 (27%)	57%
		Caucasian:	99 (44%)	51%
		Hispanic:	64 (29%)	50%

^a^% Females in FM is significantly increased as compared to % females in the Non-FM group (*p* = 1.17 × 10^− 30^)

^b^% African-Americans is significantly increased as compared to % of Caucasians in the Non-FM group (*p* = 3.11 × 10^− 12^)

**Table 2:** Adjusted odds ratios (OR) associated with each demographic feature

Demographic	Model β	Adj. OR	*P*-value
Age – vs. high (> 63 yrs)			
Mid (49–63 yrs)	0.469	1.60	1.02 × 10^− 5^
Low (< 49 yrs)	0.255	1.29	1.93 × 10^− 18^
Gender – vs. males			
Females	0.541	1.72	1.17 × 10^−30^
Ethnicity – vs. Caucasians			
African-Amer.	0.421	1.52	3.11 × 10^− 12^
Hispanic	− 0.475	0.62	3.95 × 10^− 7^
Other	− 0.635	0.53	1.78 × 10^− 6^

Logistic regression modelling the log odds of FM in the FM group compared to the non-FM group showed significant differences in age, gender, and ethnicities. Model β coefficients are the log (adjusted ORs). An individual in the mid age group, female, and African-American would have the most increased odds of FM (adjusted OR = 4.18) compared to someone who is in in the high age group, male, and Caucasian

### Frequency of COMT SNP minor alleles in FM group

**Fig. 2:**
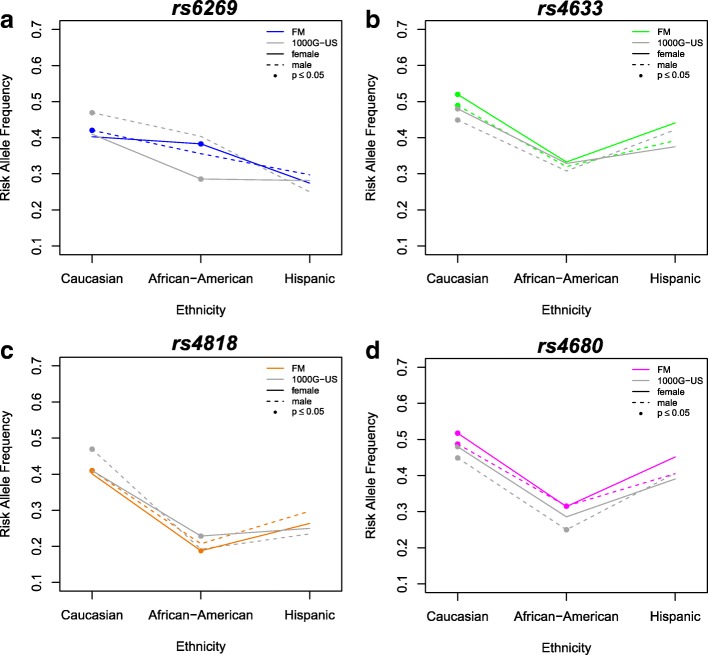
Comparing individuals with a diagnosis of fibromyalgia (FM) to US populations from the 1000 Genomes Project (1000G-US), the minor allele frequencies (MAFs) of each *COMT* SNP vary by sex and ethnicity. MAF of each *COMT* SNP are represented by colored lines in the FM group and by gray lines in the US populations from the 1000 Genomes Project. Solid lines denote females and dashed lines denote males. Significant differences (*p* ≤ 0.05) between the FM and 1000 Genomes subpopulations are represented by filled circles

Overall, the minor alleles of the *COMT* SNPs were overrepresented in the FM group compared to 1000 Genomes (Table 3). Additionally, each of the four *COMT* SNPs had significantly different minor allele frequencies (MAFs) in at least one subpopulation of the FM group than what was observed in the general population by the 1000 Genomes Project (Table 3). Differences were observed in Caucasian males, who exhibited significantly lower MAF of *rs6269*, and African-American females, who exhibited significantly higher MAF of *rs6269* (Fig. 2a). Only Caucasians exhibited significantly higher MAF of *rs4633* (Fig. 2b). Caucasian males and African-American females showed significantly lower MAF of *rs4818* (Fig. 2c). Both female and male Caucasians had higher MAF of *rs4680*. In African-Americans, only females had higher MAF of *rs4680* (Fig. 2d). MAFs of the *COMT* SNPs were not significantly different between the FM group and the non-FM group (Additional file 1: Table S1).

**Table 3:** Minor allele frequencies (MAFs) of *COMT* SNPs in subjects diagnosed with fibromyalgia (FM) compared with those of US populations from the 1000 Genomes Project (1000G-US)

SNP/Population	Minor Allele Frequency				
	1000G-US	FM Group	Direction	Model β	*P*-value
*rs6269* (A > G)	0.36	0.39	↑	− 0.03	2.40 × 10^− 6^
African-American	0.34	0.38	↑	− 0.04	0.023
Caucasian	0.44	0.41	↓	0.03	1.69 × 10^− 4^
Hispanic	0.27	0.28		−0.01	0.580
Females	0.34	0.39	↑	−0.05	1.69 × 10^−12^
*African-American*	0.29	0.38	↑	−0.09	6.04 × 10^−7^
*Caucasian*	0.41	0.40		0.01	0.472
*Hispanic*	0.28	0.27		0.01	0.831
Males	0.39	0.40		−0.01	0.540
*African-American*	0.40	0.36		0.04	0.145
*Caucasian*	0.47	0.42	↓	0.05	0.002
*Hispanic*	0.25	0.30		−0.05	0.347
*rs4633* (C > T)	0.41	0.47	↑	−0.06	1.08 × 10^−22^
African-American	0.32	0.33		−0.01	0.583
Caucasian	0.46	0.51	↑	−0.05	3.04 × 10^−8^
Hispanic	0.40	0.43		−0.03	0.348
Females	0.41	0.48	↑	−0.07	7.16 × 10^−20^
*African-American*	0.33	0.33		0	0.838
*Caucasian*	0.48	0.52	↑	−0.04	6.25 × 10^−5^
*Hispanic*	0.38	0.44		−0.06	0.064
Males	0.41	0.46	↑	−0.05	8.18 × 10^−5^
*African-American*	0.31	0.32		−0.01	0.695
*Caucasian*	0.45	0.49	↑	−0.04	0.009
*Hispanic*	0.42	0.39		0.03	0.601
*rs4818* (C > G)	0.32	0.36	↑	−0.04	2.15 × 10^−9^
African-American	0.21	0.19		0.02	0.185
Caucasian	0.44	0.40	↓	0.04	1.69 × 10^−5^
Hispanic	0.24	0.27		−0.03	0.245
Females	0.31	0.36	↑	−0.05	1.17 × 10^−9^
*African-American*	0.23	0.19	↓	0.04	0.024
*Caucasian*	0.41	0.40		0.01	0.356
*Hispanic*	0.25	0.26		−0.01	0.672
Males	0.33	0.36	↑	−0.03	0.006
*African-American*	0.19	0.21		−0.02	0.573
*Caucasian*	0.47	0.41	↓	0.06	1.19 × 10^−4^
*Hispanic*	0.23	0.30		−0.07	0.201
*rs4680* (G > A)	0.39	0.47	↑	−0.08	5.62 × 10^−31^
African-American	0.27	0.31	↑	−0.04	0.006
Caucasian	0.46	0.51	↑	−0.05	1.36 × 10^−7^
Hispanic	0.40	0.44		−0.04	0.187
Females	0.40	0.48	↑	−0.08	4.82 × 10^−23^
*African-American*	0.29	0.31		−0.02	0.145
*Caucasian*	0.48	0.52	↑	−0.04	1.67 × 10^−4^
*Hispanic*	0.39	0.45		−0.06	0.088
Males	0.39	0.46	↑	−0.07	4.35 × 10^−8^
*African-American*	0.25	0.32	↑	−0.07	0.025
*Caucasian*	0.45	0.49	↑	−0.04	0.012
*Hispanic*	0.41	0.41		0	0.988

Comparisons that are statistically significant (*p* ≤ 0.05) are indicated with direction of increased or decreased frequency of MAF. Model β coefficients are the effect sizes. Differences between groups delineated by both sex and ethnicity are graphically shown in Fig. 2

### Low and high pain sensitivity COMT diplotypes in study participants

**Fig. 3:**
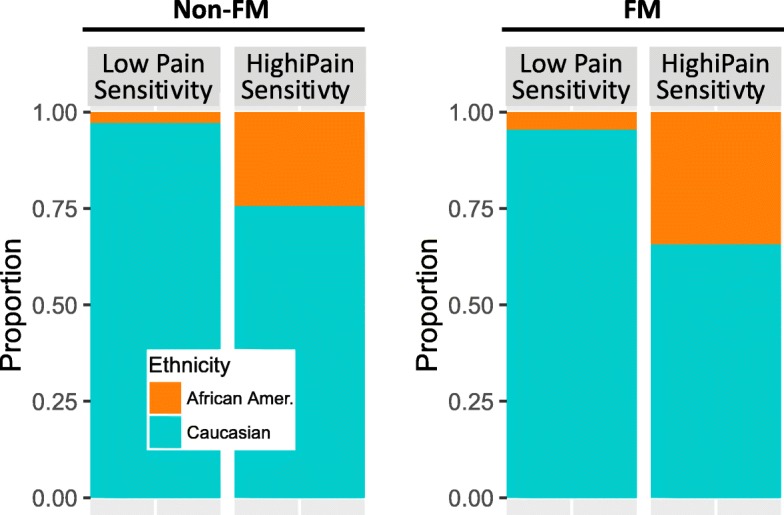
Differences in observed *COMT* diplotypes in African-Americans and Caucasians. Among those diagnosed with FM, African-Americans had 11.3 times increased odds of having a *COMT* diplotype corresponding to high pain sensitivity than low pain sensitivity, regardless of whether or not they were diagnosed with FM (*p* = 7.27 × 10^− 249^, adjusted for sex and age)

*COMT* haplotypes correspond to low (LPS), average (APS), or high (HPS) pain sensitivity phenotypes due to differences in COMT enzymatic activity [32]. Other haplotypes were also observed, but at low frequencies, did not have statistically significantly high haplotype likelihoods and were not associated with pain sensitivity in the Diatchenko et al. study [32]. In total, 2409 (89%) of FM patients and 29,170 (91%) control patients had diplotypes in combination of the three major *COMT* haplotypes: LPS/LPS, LPS/APS, APS/APS, LPS/HPS, APS/HPS, and HPS/HPS (Additional file 2: Figure S1). There were no statistically significant associations of *COMT* haplotypes or diplotypes with FM diagnosis in the FM group compared to the non-FM group. There was an association of *COMT* haplotypes or diplotypes with ethnicity groups: African-Americans were 11.3 times more likely to have *COMT* diplotype corresponding with high pain sensitivity than Caucasians, regardless of whether or not they were diagnosed with FM (*p* = 7.27 × 10^− 249^; Fig. 3).

## Discussion

Studies examining the association between COMT and fibromyalgia have largely focused on a single functional polymorphism, *rs4680* or *val*^*158*^*met*, [17, 18, 21] which reduces enzymatic activity by 3- to 4-fold, and is associated with an increased risk of chronic pain [19, 23, 40–44]. Our study demonstrates that the minor allele of *rs4680* occurs in higher frequency in FM subjects compared with population controls from the 1000 Genomes project. However, the SNP was not associated with a diagnosis of FM in this study. Other meta-analyses have both shown and refuted associations between this SNP, indicating additional variables exist that influence the susceptibility to FM. Therefore, we evaluated the association of fibromyalgia with a multi-SNP haplotype, evidenced to be more predictive of COMT enzymatic activity, and associated with a > 20-fold difference. Common COMT haplotypes were not specifically associated with FM either, suggesting that COMT enzymatic activity and catecholamine levels, per se, are not driving the development of fibromyalgia pain. We did note that while none of the common pain sensitivity-associated *COMT* haplotypes were associated with FM, the presence of minor alleles of all four of the COMT SNPs examined herein (ie: rs4680 A + rs6269 G + rs4633 T + rs4818 G is not one of the commonly described COMT haplotypes) was associated with an FM diagnosis, but would be rare.

Rather, this study shows the risk of developing FM was based on interactions with sex and ethnicity: the presence of one or more copies of a *COMT* SNP minor allele is increased in those with FM in certain demographics of ethnicity and sex. African-Americans had higher risk of a FM diagnosis than Caucasians or Hispanics, and African-American women had the highest risk overall. African-American men and women were also 11 times more likely than their Caucasian counterparts to have a *COMT* diplotype corresponding to high pain sensitivity regardless of whether or not they were diagnosed with FM. Therefore, *COMT* SNPs may provide information on the likelihood of FM when used in combination with other clinically-validated measures, such as tender points, and, as demonstrated in this study, phenotypic measures, such as ethnicity and sex. This information can be used clinically to more accurately diagnose FM early, instead of a diagnosis of exclusion.

More studies are needed to elucidate the relationship between pain sensitivity and centralized pain disorders, such as FM, but the results of this study – the largest of its kind – support a multifactorial evaluation for FM diagnosis, including ethnicity. Interestingly, the criteria used to diagnose fibromyalgia were developed in a cohort that was predominately comprised of Caucasian females [45]; this study emphasizes the need for refinement with regard to sex and/or ethnicity. However, our study relies on ICD codes to classify FM patients. Additional criteria such as tender points on patients, and detailed medical history would help to improve identification of those who truly have FM and those studies are underway. While every effort was made to exclude those with chronic pain conditions, comorbid conditions and undiagnosed FM from the control group, it is possible that such an individual who was being treated for acute pain may have been included in the control group. Thus, a clearly defined group of individuals without FM or comorbidities, such as chronic pain, would serve as a more definitive control for the FM group. Overall, our study can be strengthened with an increased number of participants and stricter criteria for FM diagnosis.

## Conclusions

This is the largest study to date to examine the role of genetics, sex and ethnicity in FM. This study builds upon previous work demonstrating an association between the *COMT* gene and chronic pain conditions. Setting it apart, however, is the multi-ethnic nature of the cohort, which explores the association between African-American ancestry, high pain sensitivity and FM diagnosis. This information can be used to refine clinical diagnostic criteria, provide information that allows clinicians to treat FM as a first diagnosis, decreasing time between the appearance of symptoms and diagnosis, and inform future studies of the importance in considering sex and ethnicity when conducting genetic association studies.

## Additional files

Additional file 1: **Table S1.** Overall minor allele frequencies of COMT SNPs in FM, 1000 Genomes, and non-FM groups. (DOCX 13 kb)

Additional file 2: **Figure S1.** Distribution of *COMT* diplotypes among FM and non-FM groups. (DOCX 69 kb)

## Abbreviations

APS: Average pain sensitivity
COMT: Catechol-O-methyltransferase
FM: Fibromyalgia
HPS: High pain sensitivity
ICD: International Statistical Classification of Diseases and Related Health Problems
LPS: Low pain sensitivity
SNP: Single nucleotide polymorphism
