# Supplementary material for: Association of Catechol-O-methyltransferase single nucleotide polymorphisms, ethnicity, and sex in a large cohort of fibromyalgia patients
Source: BMC Rheumatol. 2018 Dec 12;2:38. doi: 10.1186/s41927-018-0045-4 (PMC6390547; doi:10.1186/s41927-018-0045-4)
Supplement: Supplementary file 1 — Table S1. Overall minor allele frequencies of COMT SNPs in FM, 1000 Genomes, and non-FM groups. (DOCX 13 kb) [file 41927_2018_45_MOESM1_ESM.docx]

Table S1. Overall minor allele frequencies of *COMT* SNPs in FM, 1000 Genomes, and non-FM groups.

| *COMT* SNP | FM | Non-FM | 1000 Genomes |
| --- | --- | --- | --- |
| rs6269 | 0.39 | 0.40 | 0.36 |
| rs4633 | 0.47 | 0.47 | 0.37 |
| rs4818 | 036 | 0.37 | 0.30 |
| rs4680 | 0.47 | 0.48 | 0.37 |
